# Can physical exercise assist in controlling and reducing the severity of exercise‐induced bronchospasm in children and adolescents? A systematic review

**DOI:** 10.1111/crj.13559

**Published:** 2022-12-04

**Authors:** Bruno Rafael Vieira Souza Silva, Gerlayne Alessandra Soares da Silva, Edil de Albuquerque Rodrigues Filho, Décio Medeiros Peixoto, Camila Matias de Almeida Santos, Polyanna Guerra Chaves Quirino, José Ângelo Rizzo, Marco Aurélio de Valois Correia Junior

**Affiliations:** ^1^ Universidade de Pernambuco–UPE Recife Brazil; ^2^ Centro Universitário Mauricio de Nassau–UNINASSAU Caruaru Brazil; ^3^ Universidade Federal de Pernambuco–UFPE Vitória de Santo Antão Brazil; ^4^ Universidade Federal de Pernambuco–UFPE Recife Brazil

**Keywords:** asthma, diagnosis, exercise‐induced bronchospasm, physical exercise

## Abstract

**Objective:**

The aim of this study was to analyze whether physical exercise can contribute to improving the control and severity of exercise‐induced bronchospasm (EIB) in children and adolescents.

**Method:**

This is a systematic review that used PubMed/Medline and Scopus databases as a search source, and using descriptors indexed to DeCS/Mesh. The articles were analyzed in three stages in the selection process. Methodological quality was assessed using the TESTEX scale.

**Result and discussion:**

A total of 5867 articles were filtered in the initial search; however, only eight of these were included after the eligibility criteria. All presented improvements in cardiorespiratory fitness. Only two followed the international EIB diagnostic guidelines. Of these, only one described a reduction in FEV1 and considered that this improvement may influence the EIB response in children and adolescent athletes with a non‐asthmatic sample.

**Conclusion:**

The studies analyzed in this review did not enable drawing a conclusion regarding the influence of physical exercise on EIB in asthmatics. The lack of clinical trials on EIB and physical exercise, as well as the difficulty in methodological standardization for EIB diagnosis evidence the lack of scientific knowledge in this area, serving as a stimulus for researchers to find more consolidated answers.

## Graphical abstract

Understanding the benefits of physical exercise in adolescents with EIB can provide treatment evolutions by changing the course of the disease status. There are reports of these benefits in the literature; however, due to the small number of trials, we cannot reach a concrete conclusion.

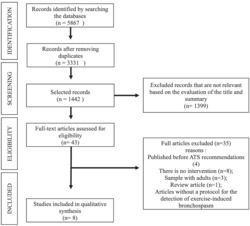

## 1 INTRODUCTION

The concept of physical activity is historically documented in the literature, being defined as any movement produced by skeletal muscle, which results in expending energy above resting levels.^1^ On the other hand, physical exercise is characterized as planned, organized and repetitive movements with the objective of maintaining, developing or recovering one or more components of physical fitness.^2^ Its continuous practice helps in the non‐pharmacological treatment of several pathologies, including asthma.^3^

Asthma is a chronic disease, common in childhood and adolescence.^4^ It is characterized by inflammation of the airways and defined by the presence of respiratory symptoms such as wheezing, dyspnoea, chest tightness, and coughing associated with limited expiratory flow.^5^ People with asthma may show less tolerance to physical exercise due to the worsening of asthma symptoms during practice, which may lead to episodes of exercise‐induced bronchospasm (EIB).^6^

EIB is defined as an acute, transient, reversible narrowing of the airways, which occurs during or after physical exercise and can be seen in patients with and without asthma.^7^ There is low agreement between young people's perception of the presence of EIB and its diagnosis, and specific pulmonary function tests are recommended for better monitoring and treatment.^8^ Since 1999, the guidelines of the American Thoracic Society (ATS) define the diagnosis of EIB as a ≥10% drop in Forced Expiratory Volume in the first second (FEV_1_) compared to baseline after standardized exercise testing with physical exercise.^9^, ^10^

A recent systematic review pointed to a 46% prevalence of EIB in children and adolescents with asthma in Europe, Africa, Asia‐Pacific, and America, estimating that about 16.5 million of this population up to 18 years of age may develop EIB,^11^ and with that impair their practice of activities and games in such an important phase of life, which is fundamental for physical, psychological, and mental development. Although physical exercise is a triggering agent for EIB, its practice is reported as a great ally in treating asthma in children and adolescents,^12^ in addition to the already known effects of improvements in cardiorespiratory fitness, reduction of post‐exercise dyspnea and improved quality of life.^13^, ^14^, ^15^

Because it is a limiting factor for involvement and practice of physical exercise, understanding the ways of diagnosing EIB and intervention with physical exercise in children and adolescents may contribute to providing guidelines to more assertively practice physical exercise in this population.^10^, ^16^ In this sense, the objective of this study was to analyze whether physical exercise can contribute to improving EIB control and severity in children and adolescents.

## 2 METHODOLOGICAL PROCEDURES

The present study is a systematic review of the literature searching the PubMed/Medline and Scopus databases in August 2020 and updated in January 2021. The search for descriptors and terms was performed with MeSh (Medical Subject Headings) and DeCS (Health Sciences descriptors), associated with Boolean descriptors. The search descriptors used were: “*Children*” *AND* “*Adolescent*” *AND* “*Exercise‐induced asthma*” OR “*Exercise‐induced bronchospasm*” *AND* “*Exercise*” *OR* “*Physical exercise*,” and they should appear at least in the title, abstract or keywords of the articles.

The PICO strategy was employed in the present systematic review (*P*atients: Children or Adolescents with EIB; *I*ntervention: Physical exercise; *C*omparisons: pre and post exercise; *O*utcome: Control or alteration of EIB after systematic intervention with physical exercise). Studies in English and Portuguese published since 1999 were selected according to the American Thoracic Society (ATS) guidelines for testing with methacholine and challenge with exercise.^9^ The search and analysis of the articles were independently conducted by two evaluators (BRVSS and GASS) according to the previously established strategies, with the differences resolved by a third evaluator (MAVCJ).

**FIGURE 1:**
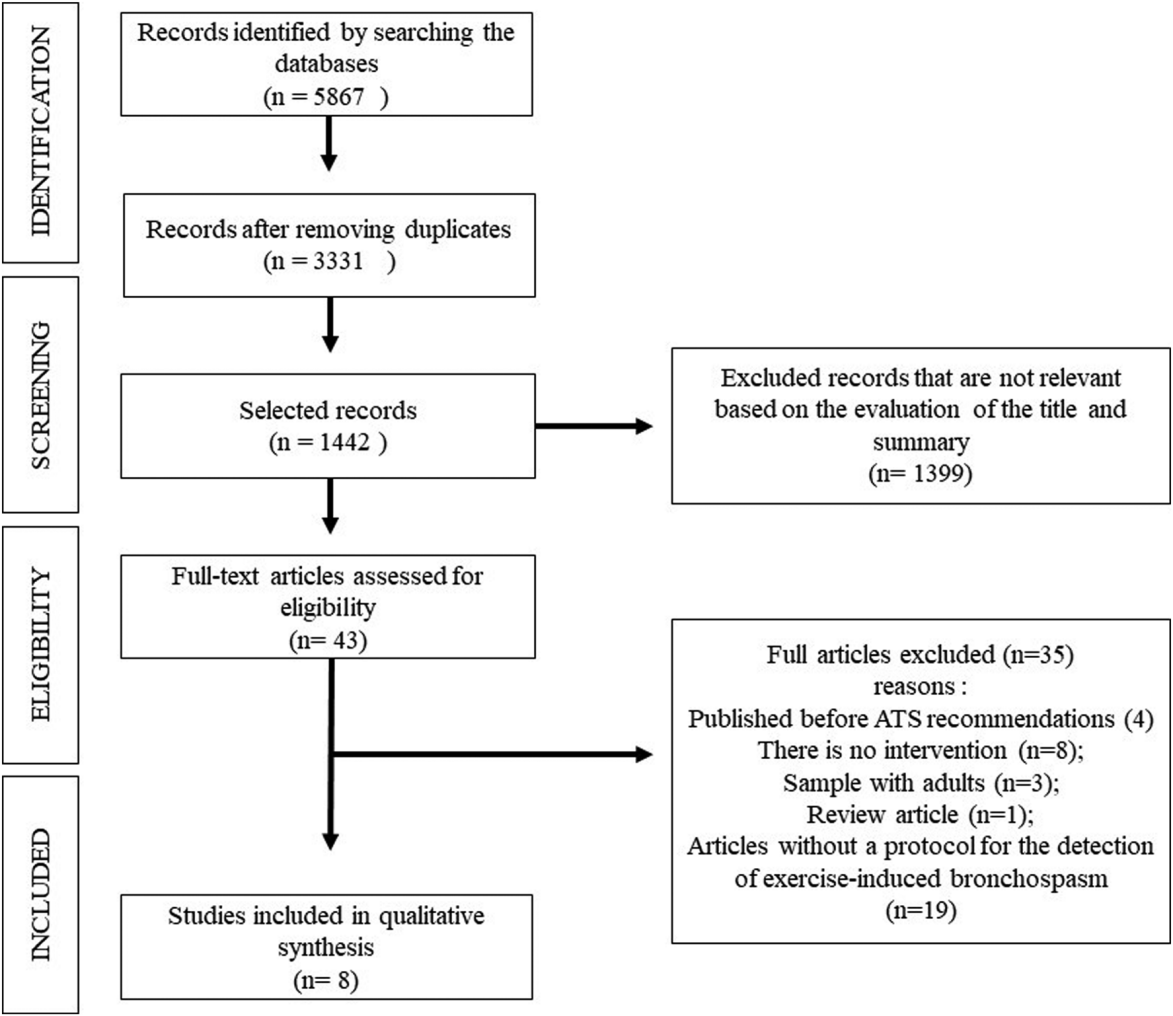
Flowchart of the steps of the search process and selection of the articles included. **Source:** Own authorship.

The following inclusion criteria were used: (1) clinical trials conducted with children and/or adolescents with EIB; (2) presence of protocols for detecting EIB^7^, ^9^; (3) employed some physical exercise or systematic physical training; (4) the training intervention lasted at least 4 weeks following the criteria used in the Cochrane systematic review on physical activity for asthma^6^; (5) presented comparisons with a control group. Studies that investigated: animals; subjects without EIB; combinations of asthma with other diseases; studies that only performed breathing exercises; revisions; guidelines; cards; brief comments; book chapters; conference abstracts; monographs; dissertations and theses were excluded. The titles and abstracts of the identified studies were assessed against eligibility criteria and were read in full only after approval of the criteria. Figure 1 shows the flowchart containing the search and selection process stages for the articles included in this systematic review.

The following variables were searched and systematically extracted from among the eligible articles, and then inserted in a summary table: (1) authors; (2) year of publication; (3) age group; (4) sample size; (5) evaluation and diagnosis form of EIB; (6) details of the physical exercise program: time, type, intensity, duration, and frequency; (7) main results.

We used the “*T*ool for the ass*E*ssment of *S*tudy quali*T*y and reporting in *EX*ercise” (TESTEX)^17^ to assess the methodological quality of the articles included, which establishes a formal quality score for each study on a 15‐point scale, assigning a value of 0 for absent or inadequately described, or 1 for present and explicitly described for the following questions: (1) specified eligibility; (2) specified randomization; (3) concealment of allocation; (4) similar groups at the beginning of the study; (5) masking the evaluator; (6) outcome measures evaluated in >85% of the sample; (7) reported adverse events; (8) attendance to the reported exercise; (9) used intention‐to‐treat analysis; (10) statistical comparison between groups; (11) data variability for all outcomes; (12) monitoring of the control group; (13) maintaining the physical exercise intensity; (14) exercise volume; (15) reported energy expenditure. Based on the assigned scores, we classified the studies as “excellent quality” (12 to 15 points), “good quality” (9 to 11 points), “reasonable quality” (6 to 8 points), and “low quality” (<6 points). Two authors (BRVSS and GASS) independently conducted the quality assessments and any disagreements were resolved through discussions and agreements (Table 1).

**TABLE 1:** Quality assessment using the checklist TESTEX

First author	Evaluation criteria TESTEX															
	1	2	3	4	5	6	7	8	9	10	11	12	13	14	15	Total
Nedder	*1*	*0*	*0*	*1*	*0*	*1*	*1*	*1*	*1*	*1*	*1*	*0*	*1*	*1*	*0*	**10**
Matsumoto	*1*	*1*	*0*	*1*	*0*	*0*	*0*	*1*	*0*	*1*	*1*	*0*	*1*	*1*	*1*	**9**
Veldhoven	*1*	*1*	*0*	*1*	*0*	*1*	*0*	*0*	*1*	*1*	*1*	*0*	*0*	*1*	*0*	**8**
Natali	*1*	*1*	*1*	*1*	*0*	*0*	*0*	*0*	*1*	*1*	*1*	*1*	*1*	*1*	*1*	**11**
Silva	*1*	*1*	*0*	*1*	*0*	*1*	*0*	*0*	*0*	*1*	*1*	*0*	*1*	*1*	*1*	**9**
Fanelli	*1*	*1*	*0*	*1*	*0*	*1*	*1*	*1*	*1*	*1*	*1*	*0*	*1*	*1*	*1*	**12**
Sindiropoulou	*1*	*1*	*0*	*1*	*0*	*0*	*1*	*0*	*1*	*1*	*1*	*1*	*1*	*1*	*1*	**11**
Tahan	*1*	*1*	*0*	*1*	*0*	*1*	*0*	*1*	*0*	*1*	*1*	*0*	*0*	*1*	*0*	**8**

Abbreviations: 0, criteria not met; 1, criteria met.

## 3 RESULTS

Figure 1 shows the flowchart of the study screening process, resulting in 5867 titles based on the initial search. After removing duplicates and analyzing titles and abstracts, 43 articles remained to be reviewed and analyzed in full; then after applying the inclusion and exclusion criteria, eight of these were considered to be potentially relevant for the present review. Of the articles excluded for not meeting the inclusion criteria, four of them (Fitch et al.,^18^ Orenstein et al.,^19^ Jain et al.,^20^ and Olivia et al.^21^) were published before the ATS guidelines,^7^, ^9^ and therefore, they presented disagreements in relation to the EIB detection test protocol standardized by this entity.

The data related to the characteristics of the selected articles are shown in Table 2. All studies were characterized as a randomized clinical trial, and only one did not obtain a homogeneous sample at the beginning of the investigation (baseline FEV_1_ was different between the control and the intervention group at the beginning of the intervention).^22^ The publications varied between the years of 1999^22^ to 2014.^23^ Three studies (Neder et al.,^24^ Veldhoven et al.,^25^ and Silva et al.^26^) did not explicitly have the main objective to analyze physical exercise and its influence on EIB. The age of the sample varied between 6 and 17 years, being composed of children and/or adolescents with asthma, except for one study (Sidiropoulou et al.^27^), which had a sample of individuals with EIB, without diagnosed asthma.^27^ Regarding the EIB detection protocol, only two studies (Silva et al.^26^ and Sidiropoulou et al.^27^) followed all the recommendations presented in the ATS. The greatest difficulties encountered in classifying the EIB diagnosis were related to post‐exercise spirometry as identified in Table 2. All patients underwent spirometry before and after exercise, and three studies (Matsumoto et al.,^28^ Veldhoven et al.,^25^ and Natali et al.^29^) did not reassess the participants after 20 minutes of the exercise test.

**TABLE 2:** Sample characteristics and aspects related to the diagnosis of exercise‐induced bronchospasm

Author, place and year	EIB on objective	Sample			Protocol for EIB diagnosis										
		*N*	Age	Public	Bronchial provocation test performed	Exercise intensity	Post‐test evaluation								Diagnostic
							<5′	5′	7,5′	10′	15′	20′	30′	45′	
Neder et al. 1999^24^ Brazil^a^	No	24	6 à 17 years	Moderate to severe asthma	Pré: Spirometry—6‐min test on the treadmill	80% MCF (220‐age). 1 min to get max.		X		X		X			∆FEV_1_ > 10%
Matsumoto et al. 1999^28^ Japan^b^	Yes	16	8 à 12 years	Asthmatics	Pré: Spirometry—6‐min swim test and cycle ergometer	175% and 100% of the lactate threshold.		X			X				∆FEV_1_ > 10%
Veldhoven et al. 2001^25^ Netherlands^c^	No	47	8 à 13 years	Moderate to severe asthma	Pré: Spirometry—6‐min test on the treadmill	20–30 beats lower than predicted (220‐age)	X		X	X					∆FEV_1_ > 10%
Natali^29^ et al. 2002 Brazil^d^	Yes	32	10 à 16 years	Asthmatics	Pré: Spirometry—6‐min test on the treadmill	85%–90% MCF (220‐age). 2 min to get max.		X							∆MEF_1_ > 10%–15%
Silva^26^ et al. 2006 Brazil	No	69	8 à 11 years	Asthma Moderate/persistent	Pré: Spirometry—6‐min test on the treadmill	80% MCF (220‐age) 1 min to get max.		X		X	X	X	X	X	∆FEV_1_ > 10%
Faneli^30^ et al. 2007 Brazil^e^	Yes	38	7 à 15 years	Moderate to severe asthma	Pré: Spirometry—6‐min test on the treadmill	80% MCF. 4 min to get max.		X		X		X			∆FEV_1_ > 10%
Sidiropoulou^27^ et al. 2007 Greece	Yes	29	9 à 16 years	Athletes with EIB	Pré: Spirometry—6‐min test on the treadmill	80%–90% MCF.	X	X		X	X	X	X		∆FEV_1_ > 10%
Tahan^23^ et al. 2014 Turkey^f^	Yes	24	6 à 17 years	Moderate asthma	Pré: Spirometry—6‐min test on the treadmill	90% MCF (220‐age) 1 min to get max.		X		X	X	X			∆FEV_1_ > 15%

*Note*: Superscript lines indicate possible limitations found in the methodology of the studies found. MCF = Maxima Cardiac Frequency. MEF = Maximum Expiratory Flow. FEV1 = Forced Expiratory Volume in the first second. Taxed = studies that followed the recommendations for exercise‐induced bronchospasm (EIB) assessment.

^a^ Study analyzes groups with a non‐homogeneous sample in the selection of subjects and in FEV_1_.

^b^ Effort prescription was by lactate threshold.

^c^ Does not show spirometry values after 10 min.

^d^ Diagnosis of EIB through maximum expiratory flow (MEF).

^e^ Does not show spirometry after 20 min.

^f^ Study does not define its control group adequately.

Table 3 presents information related to physical exercise interventions. In all, 168 subjects who participated in the intervention groups followed some systematic physical exercise protocol. Only three studies (Faneli et al.,^30^ Sidiropoulou et al.,^27^ and Tahan et al.^23^) performed an intervention with the control group, namely: educational intervention,^30^ traditional training method,^27^ and yoga practice.^23^ Regarding the training variables, the study time lasted between six and 16 weeks, the duration of the sessions ranged from 30 to 90 min and the weekly frequency was between two to six times a week. Regarding the intervention intensity, three studies (Neder et al.,^24^ Vendhoven et al.,^25^ and Tahan et al.^23^) did not present the forms of intensity monitoring in their procedures. All the analyzed articles pointed out improvements in cardiorespiratory fitness in the intervention group, and five investigations^23^, ^27^, ^28^, ^29^, ^30^ reported significant results in relation to reducing EIB (without taking into account issues related to the diagnostic method).

**TABLE 3:** Characteristics of the intervention procedures and results obtained by the studies analyzed

Author/year	Group division	Intervention group			Group control	Results	Conclusions
		Type	Time	Intensity			
Neder et al. 1999	Intervention: 26 Control: 16	Treadmill and exercise bike (Aerobic)	8 weeks; 45 min; 3 times/week;	Not defined	He did not perform any intervention.	↑ Cardiorespiratory fitness ↔ % FEV_1_–EIB	Short‐term physical exercise improves physical fitness but does not influence EIB.
Matsumoto et al. 1999	Intervention: 8 Control: 8	Swimming (Aerobic)	6 weeks; 30 min; 6 times/week;	125% do lactate threshold.	He did not perform any intervention.	↑ Cardiorespiratory fitness **↓ % FEV**_**1**_–**EIB**	The drop in FEV1 points to physical exercise as a protective factor for EIB.
Veldhoven et al. 2001	Intervention: 23 Control: 24	Sports training (Concurrent exercise)	12 weeks; 60 min; 2 times/week;	Not defined	He did not perform any intervention.	↑ Cardiorespiratory fitness ↔ % FEV_1_–EIB	The occurrence of EIB before and after the intervention did not differ between groups.
Natali et al. 2002	Intervention: 16 Control: 16	Swimming (Aerobic)	10 weeks; 45 min; 3 times/week;	85% da MCF	He did not perform any intervention.	↑ Cardiorespiratory fitness **↓ % FEV**_**1**_–**EIB**	It is suggested that the increase in FVC and MEF is indicative of a reduction in EIB.
Silva et al. 2006	Intervention: 46 Control: 23	Functional circuit (Concurrent exercise)	12 weeks; 90 min; 2 times/week;	Individually monitored	He did not perform any intervention.	↑ Cardiorespiratory fitness ↔ % FEV_1_–EIB	FEV1 values did not change before and after the intervention.
Faneli et al. 2007	Intervention: 21 Control: 17	Aquatic and terrestrial (Concurrent exercise)	16 weeks; 90 min; 2 times/week;	Individually monitored	Educational program: ABC of asthma.	↑ Cardiorespiratory fitness **↓ % FEV**_**1**_–**EIB**	Improvement in aerobic fitness and control of dyspnea if a reduction in EIB is obtained.
Sidiropoulou et al. 2007	Intervention: 18 Control: 11	Soccer (Interval exercise)	8 weeks; 60 min; 3 times/week;	80%–90% da MCF	Conventional football exercise without control.	↑ Cardiorespiratory fitness **↓ % FEV**_**1**_–**EIB**	Systematic exercise program avoids episodes of EIB.
Tahan et al. 2014	Intervention: 10 Control: 10^e^	Yoga	12 weeks; 60 min; 2 times/week;	Not defined	Practiced yoga	↑ Cardiorespiratory fitness **↓ % FEV**_**1**_–**EIB**	The practice of physical exercise has beneficial effects on EIB.

*Note*: MCF = Maxima Cardiac Frequency ↑ increase; ↓ reduction; ↔ No changes. FEV_1_ = Forced Expiratory Volume in the first second. ^e^ = Control Group was asthmatic children without EIB. Taxed = studies that followed the recommendations for EIB assessment.

## 4 DISCUSSION

The main objective of this systematic review was to analyze whether physical exercise can contribute to improving EIB control and severity in children and adolescents. Of the eight studies selected to compose our results, five presented EIB as the main objective and only two followed the internationally recognized recommendations for the diagnosis of EIB.^26^, ^27^ Of these, only one study^27^ pointed out a significant improvement in reducing FEV_1_ and considered that this improvement may influence the response to EIB in children and adolescent athletes with a non‐asthmatic sample. In the other study, the EIB assessment was only performed to characterize the sample and not as the main objective. Even so, the authors compared FEV_1_ before and after intervention between groups, with no statistical difference.^26^ The difficulty found in the search for studies, the great methodological diversity identified (both for the diagnostic classification of EIB and for the prescription of physical exercise), in addition to the lack of studies with an objective focus on this theme exposes the need for greater awareness among researchers about the contribution of physical exercise in controlling asthma symptoms and EIB.

The benefits of physical exercise in general and in asthma, unlike its relationship with EIB, are well documented in the scientific literature worldwide.^7^, ^14^, ^31^, ^32^ The Global Strategy for Asthma Management and Prevention (GINA),^31^ recommends practicing physical exercise as part of non‐pharmacological treatment aiming at general health benefits, as well as in EIB management by associating exercise with appropriate medication. Physical exercise programs aimed at chronic diseases including asthma show improvement in physical fitness and add an important effect in improving psychosocial aspects.^33^ In addition, exercise has a modulating role in pulmonary inflammation, in reducing bronchial hyperresponsiveness and the need to use corticosteroids (inhaled or oral), as well as a decrease in the number of eosinophils in sputum and in the levels of exhaled nitric oxide.^34^, ^35^, ^36^ Clinical trials show that a physical training program also improves anxiety and depression symptoms, as well as quality of life in people with asthma.^32^, ^37^, ^38^

The ATS^7^, ^9^ recommends that the exercise duration to diagnose EIB is 8 to 10 min, keeping the heart rate between 80% and 90% of the maximum calculated for age during the last 6 min of effort. FEV_1_ measurements using spirometry should be performed serially before and at 5, 10, 15, and 30 min after exercise, with a reduction greater than or equal to 10% in FEV_1_ compared to baseline being considered positive.^9^, ^39^ There were two studies^26^, ^27^ that followed the recommendations for the EIB diagnosis^7^, ^9^ in our findings, which points out that the vast majority of the included studies are outside the diagnosis standard and leaving the results regarding EIB reduction after performing physical exercise as questionable.

A recent systematic review^40^ in which eight studies were selected stated that there is insufficient evidence that physical exercise contributes to reduce EIB in children and adolescents with asthma, only pointing to an improvement in cardiorespiratory fitness. However, it is noteworthy that in their selected articles,^40^ adolescents were evaluated together with adults as inclusion in the article by Hallstrand et al.^41^ Another point to highlight is that, differently from our research, the results presented by the authors did not consider the diagnostic criteria for EIB pointed out by the ATS.^7^, ^9^ An example of this problem is the study by Natali et al.,^29^ who performed the EIB diagnosis through Maximum Expiratory Flow (MEF), and Matsumoto et al.^28^ who used the lactate threshold to calculate the effort intensity instead of maximum heart rate. Our study adds the study developed by Veldhoven et al.^25^ in its results, who did not perform spirometry after 10 min, in addition to not making clear the diagnosis parameters of EIB, despite talking about its severity by FEV_1_. Only two studies (Sidiropoulou et al.^27^ and Silva et al.^26^) included in this review performed spirometry at least 30 min after the bronchial challenge test. All of these highlights were exemplified in Table 2.

For this reason, we call attention to assess the methodological quality of studies related to the EIB diagnosis. We used the TESTEX scale in our review to evaluate clinical trials with physical exercise, where we noticed that most articles (62.5%) pointed out a “good methodological quality” in the studies and none had bad quality. Even so, of the eight studies chosen with good quality in TESTEX, only two^26^, ^27^ presented an adequate methodology for diagnosing EIB according to the criteria adopted in the present review.

In relation to the intervention with physical exercise, we approached the prescription of systematic physical exercise as an inclusion criterion, meaning the exercise type, time and intensity needed to be described in the articles. Among the analyzed studies, there was great variability in the modalities found (aerobic,^24^, ^28^, ^29^ concurrent exercise,^25^, ^26^, ^30^ interval training,^27^ and yoga^23^). However, only two studies that evaluated concurrent training with a functional circuit^26^ and the interval training method with soccer exercises^27^ were considered methodologically appropriate.

The American College of Sports Medicine (ACSM)^42^ points out in its publications the importance of prescribing aerobic exercises alternated with resistance exercises (concurrent method) for several populations, resulting in improvements in general physical fitness. Herein, three articles^25^, ^26^, ^30^ used the application of concurrent training resulting in improved aerobic fitness and reduced dyspnea after exercise. However, despite Faneli et al.^26^ reporting a reduction in EIB episodes, the present review identified methodological issues in the EIB diagnosis, which should be taken into account (Table 2). The studies that resorted to aerobic methods,^24^, ^28^, ^29^ even though they also reported improvement in cardiorespiratory fitness, the methodological issues relevant to this review and which did not allow adequate conclusions for improvement in physical exercise and EIB outcome were: prescription by the threshold of lactate,^28^ starting the study with a non‐homogeneous sample^24^ and only performing spirometry at 5 min after exercise for the EIB diagnosis, which could generate false negative results.

Interval training is characterized by alternating intensity from moderate to high during the effort period. Only one study used this methodology^27^ of soccer training in children and adolescent athletes who were not diagnosed with asthma. This was the only study selected with a standardized methodology with positive results in reducing FEV_1_. Evidence points to satisfactory results with regard to yoga practice in treating asthma.^43^ In our findings, the study by Tahan et al.^23^ reported a reduction in FEV_1_ inducing improvement in controlling EIB; however, the study does not present a control group, and uses the cut‐off point for the decrease in the FEV_1_ as a diagnostic criterion for the EIB >15% of baseline.

We believe that the practice of physical exercise, regardless of its modality and training method, is beneficial for cardiorespiratory improvement and that it reflects on the quality of life in the general public and should always be encouraged; however, the results shown herein demonstrate an important knowledge gap with regard to EIB in adolescents, especially asthmatics. We believe that this review can serve as an incentive for researchers in the field to carry out new research with similar methodologies regarding EIB diagnosis and that soon we can find more convincing results about exercise and EIB.

Some limitations in this study should be mentioned, such as the low number of studies found related to the theme; the age group, in which the studies included children and adolescents in different phases; the methodological variability in the studies that reflected in presenting the results and discussion of the data; and the different statistical treatments adopted. However, the results found are significant and constitute a reference for further studies on this topic and have strengths that need to be highlighted, such as: the strategy and methodological rigor adopted, the presentation of in‐depth results of the selected studies, as well as the use of scales to evaluate the quality of studies.

## 5 CONCLUSION

There is a lack of studies in the literature with information on performing physical exercise in children and adolescents with EIB, which makes it inconclusive to answer whether exercise improves EIB control and severity. The lack of clinical trials on EIB and physical exercise, as well as the difficulty in methodological standardization for EIB diagnosis evidence the lack of scientific knowledge in this area, serving as a stimulus for researchers to find more consolidated answers.

## ETHICS STATEMENT

All ethical precautions were strictly followed, with the aim of presenting content capable of contributing to the scientific community.

## CONFLICT OF INTEREST

We declare that there are no conflicts of interest.

## AUTHOR CONTRIBUTIONS

We, Bruno Rafael Vieira Souza Silva, Gerlayne Alessandra Soares da Silva, Edil de Albuquerque Rodrigues Filho, Décio Medeiros Peixoto, Camila Matias de Almeida Santos, Polyanna Guerra Chaves Quirino, José Ângelo Rizzo, and Marco Aurélio de Valois Correia Junior, are responsible for the content and authenticity of the work entitled “CAN PHYSICAL EXERCISE ASSIST IN CONTROLLING AND REDUCING THE SEVERITY OF EXERCISE‐INDUCED BRONCHOSPASM IN CHILDREN AND ADOLESCENTS? A SYSTEMATIC REVIEW,” and we declare that the referred article was never published or sent to another magazine. We also declare that all authors contributed satisfactorily from the construction of the idea to the finalization of the manuscript. BRVSS, GASS, and MAVCJ worked on the conception, design, analysis, and interpretation of data, writing of the article, critical review, and approval of the version to be submitted; EARF, DMP, and JAR participated in the critical review of data, comparison of results, analysis of variables, and spelling corrections. CMAS and PGCQ contributed to the writing, methodological path and critical review of the article.

## Data Availability

The data that guide this article are found throughout the manuscript.
